# *In Vivo* Effect of Arsenic Trioxide on Keap1-p62-Nrf2 Signaling Pathway in Mouse Liver: Expression of Antioxidant Responsive Element-Driven Genes Related to Glutathione Metabolism

**DOI:** 10.1155/2013/817693

**Published:** 2013-07-10

**Authors:** Ritu Srivastava, Archya Sengupta, Sandip Mukherjee, Sarmishtha Chatterjee, Muthammal Sudarshan, Anindita Chakraborty, Shelley Bhattacharya, Ansuman Chattopadhyay

**Affiliations:** ^1^Radiation Genetics and Chemical Mutagenesis Laboratory, Department of Zoology, Centre for Advanced Studies, Visva-Bharati University, Santiniketan, West Bengal 731235, India; ^2^Environmental Toxicology Laboratory, Department of Zoology, Centre for Advanced Studies, Visva-Bharati University, Santiniketan, West Bengal 731235, India; ^3^UGC-DAE Consortium for Scientific Research, Kolkata Centre, 3/LB-8, Bidhan Nagar, Kolkata, West Bengal 700098, India

## Abstract

Arsenic is a Group I human carcinogen, and chronic arsenic exposure through drinking water is a major threat to human population. Liver is one of the major organs for the detoxification of arsenic. The present study was carried out in mice *in vivo* after arsenic treatment through drinking water at different doses and time of exposure. Arsenic toxicity is found to be mediated by reactive oxygen species. Nuclear factor (erythroid-2 related) factor 2 (Nrf2)/Keap1 (Kelch-like ECH-associated protein 1)/ARE (antioxidant response element)—driven target gene system protects cells against oxidative stress and maintains cellular oxidative homeostasis. Our result showed 0.4 ppm, 2 ppm, and 4 ppm arsenic trioxide treatment through drinking water for 30 days and 90 days induced damages in the liver of Swiss albino mice as evidenced by histopathology, disturbances in liver function, induction of heat shock protein 70, modulation of trace elements, alteration in reduced glutathione level, glutathione-s-transferase and catalase activity, malondialdehyde production, and induction of apoptosis. Cellular Nrf2 protein level and mRNA level increased in all treatment groups. Keap1 protein as well as mRNA level decreased concomitantly in arsenic treated mice. Our study clearly indicates the important role of Nrf2 in activating ARE driven genes related to GSH metabolic pathway and also the adaptive response mechanisms in arsenic induced hepatotoxicity.

## 1. Introduction

Arsenic (As), a Group I human carcinogen, is the major source of ground water contamination all over the world. The permissible limit of As, set by World Health Organization (WHO) is 10 parts per billion (ppb). However, in many countries including India and Bangladesh, people are consuming As through drinking water at much higher level. Up to 50 ppm of As is reported in many states in the USA [1]. Fu et al. [2] estimated that 13 million Americans were exposed to more than 0.01 ppm of arsenic through public water systems by 2006. According to the report of the Prevention and Treatment Academy of China, this number reached 14.66 million in China [3] where in many places individuals were exposed up to a level of 1 ppm of As [4]. In West Bengal, India As concentrations in some tube wells is as high as 3.4 ppm [5]. Chronic arsenic exposure has become a great concern than acute exposure mainly because of its carcinogenic effects [6, 7]. Environmental exposure to arsenic is generally in the form of either arsenite (As^3+^) or arsenate (As^5+^) which undergoes redox conversion, where arsenite is the predominant form in drinking water and is considered as the major carcinogen in epidemiological studies [6, 8].

 Liver is one of the major target organs of arsenic toxicity and carcinogenesis [9–11]. When consumed through drinking water, inorganic As species is converted into its methylated form within the liver and excreted out [12]. In exposed human populations chronic arsenic causes variety of toxic effects in the liver and other organs and is associated with tumerogenesis [13]. Gastrointestinal symptoms, abnormal liver function, and elevations of serum enzymes like alanine amino transferase (ALT), aspartate amino transferase (AST), and alkaline phosphatase (ALP) are reported after acute or chronic exposure [14, 15]. Developments of portal hypertension and liver fibrosis have also been observed among As-exposed populations [14, 16]. Histopathology and induction of stress protein by arsenic have been reported in *Channa punctatus* [17]. The roles of reactive oxygen species (ROS) and reactive nitrogen activity are known during arsenic toxicity [18–20], but the exact mechanism for the generation of all these reactive species is yet to be elucidated [21]. Glutathione (GSH), the major nonprotein thiol in mammalian cells is a well known free radical scavenger and reducing equivalent and plays a major protective role against ionizing radiation as well as chemical reagents generating ROS. Liver is a rich source of GSH and a major site for arsenic detoxification, through GSH-As conjugation pathway [9]. Trivalent arsenicals readily react *in vitro* with GSH and cysteine [22]. The binding of trivalent arsenic to critical thiol groups causes GSH depletion affecting the status of other antioxidants and thus inhibits important biochemical events which could lead to toxicity [23]. However, the effect of arsenic on GSH metabolic pathway in liver *in vivo* is not yet clearly known which prompted the present investigation.

Cellular oxidative homeostasis is maintained by a transcriptional factor Nrf2 (Nuclear factor erythroid 2-related factor 2) which does so through transcriptional upregulation of an array of downstream genes, such as glutathione s-transferase (GST), glutathione peroxidase (GPx), glutathione reductase (GR), *γ*-gamma glutamyl cysteine synthase (*γ*GCS), glutamate cysteine ligase (GCL), heme oxygenase-1 (HO-1), and NAD(P)H quinone oxidoreductase-1 (NQO1) [24–26]. Studies on As-induced activation of Nrf2 and its downstream genes have been reported in different cell lines [20, 27–32]. Nrf2 plays a pivotal role in modulating the expression of phase II detoxification enzymes and endogenous antioxidants. Using Nrf2 knockout mice, Jiang et al. [12], showed that Nrf2 protects against liver and bladder injury in response to six weeks of arsenic exposure, but the detailed mechanism, particularly its role on the GSH-metabolic pathway *in vivo*, was not studied.

In some parts of West Bengal, India, arsenic concentration is reported to be as high as 3.4 ppm [5]. Therefore we selected three doses (0.4, 2, and 4 ppm of As_2_O_3_) through drinking water in mice, to see their effect on body weight gain, organ to body weight ratio, and histopathology of liver after chronic exposure for one to three months. Level of GSH, activity of GST and catalase, MDA production, and expression of Hsp70, Nrf2, Keap1 (Kelch-like ECH-associated protein 1), p62 and ARE driven genes for antioxidant enzymes involved in GSH-metabolic pathway were observed to understand the role of Nrf2 on As-induced hepatotoxicity.

Trace element profile is a valuable marker for health status of animal body, and any disturbance in the profile indicates malfunctioning of the normal metabolism. Trace elements are involved in almost every cellular biochemical process, and inadequacy or imbalance in the level of trace element consequently affects a number of physiological functions. The modulation of some important trace elements that mediate oxidative stress and are related to redox status of the cells such as copper (Cu), zinc (Zn), iron (Fe), magnesium (Mg) and selenium (Se) was considered in the present study since they are the key elements in cellular protection against As-induced hepatic damages which could influence the Nrf2 mediated antioxidant responses.

## 2. Materials and Methods

### 2.1. Chemicals and Reagents

Arsenic trioxide (As_2_O_3_, molecular weight 197.84) was purchased from Sigma-Aldrich Corp. (St. Louis, MO, USA). Glutamate oxaloacetate transaminase GOT (AST) and glutamate pyruvate transaminase GPT (ALT) test kits were purchased from Span diagnostics Ltd., Surat, India. Primary antibodies against Hsp 70, Nrf2, gamma glutamyl cysteine synthase (*γ*GCS), glutathione reductase (GR), glutathione S-transferase (GST), p62, Keap1, *β*-actin were purchased from Santa Cruz Biotechnology Inc. (Santa Cruz, CA, USA). Mouse anti-rabbit ALP conjugated secondary antibody, Hoechst (Bisbenzimide H 33342), BCIP/NBT, and TRI reagent for RNA isolation were procured from Sigma-Aldrich Corp. (St. Louis, MO, USA). Reverse transcriptase and all chemicals of PCR mix were purchased from Fermentas (USA). All other chemicals used were of analytical grade and purchased from Sisco Research laboratories (Mumbai, India) and Merck (Darmstadt, Germany). 

### 2.2. Animals and Treatment

Male Swiss albino mice, aged 2-3 months, weighing 25–30 g, were maintained in community cages in a temperature-controlled room at 20 ± 2°C and 12 hr light/12 hr dark cycle. They were fed standard mouse diet procured from NMC Oil Mills Ltd, Pune, India and were provided with water *ad libitum.* Animal studies were approved by the Institutional Animal Ethics Committee, Visva-Bharati University and were performed in accordance with the ethical standards laid down in the 1964 Declaration of Helsinki and its later amendments. Mice were divided into five groups with 6 mice per group as given below. All mice were sacrificed under anesthesia using light sodium pentobarbital:

 Group I: untreated animals (control),
 Group II: 0.4 ppm As_2_O_3_ treated through drinking water for 30 days,
 Group III: 2 ppm As_2_O_3_ treated through drinking water for 30 days,
 Group IV: 4 ppm As_2_O_3_ treated through drinking water for 30 days,
 Group V: 4 ppm As_2_O_3_ treated through drinking water for 90 days.

### 2.3. Measurement of Body Weight and Water Consumption

The body weight of all animals was recorded initially and also during the course of the treatment. Rate of water consumption and gain in body weight were recorded for each individual mouse at certain time intervals during the experiment.

### 2.4. Determination of Organ to Body Weight Ratio

The weight of the mice was recorded before sacrifice. The liver was dissected out carefully, blotted free of blood, and fresh weight was recorded. Organ to body weight ratio was calculated and compared with the control mice.

### 2.5. Histopathological Studies

Portions of liver tissue of all animals were fixed in Bouin's fluid, dehydrated through graded alcohol, and embedded in paraffin, and routine microtomy was carried out to obtain 6 *μ*m thick tissue sections. Sections were stained by routine hematoxylin-eosin (HE) technique and viewed under light microscope.

### 2.6. Liver Function Tests

Serum glutamate oxaloacetate transaminase (SGOT) (aspartate transaminase; AST) and serum glutamate pyruvate transaminase (SGPT) (alanine transaminase; ALT) levels were estimated following the manufacturer's protocol.

### 2.7. Determination of Reduced Glutathione (GSH)

Liver GSH was measured following the method of Beutler et al. [33]. In brief, liver tissue was quickly dissected out and blanched in ice-cold isotonic saline. A 10% homogenate was prepared from each tissue with ice-cold saline-EDTA at 4°C. One milliliter of freshly prepared 20% ice-cold trichloroacetic acid (TCA) was added to equal volume of homogenate, and the mixture was vortexed and allowed to stand for 10 min in 4°C. The mixture was then centrifuged at 5,000 rpm for 5 min. The clear supernatant was used as the GSH sample from which 1 mL of supernatant was taken and mixed with 3 mL of 0.3 M disodium hydrogen phosphate buffer and 1 mL of 5,5′-dithiobis-2-nitro benzoic acid (DTNB) solution. After 5 min, the optical density of the samples was measured at 412 nm, and results were expressed as *μ*M GSH/mg protein.

### 2.8. Assay of Glutathione-s-Transferase (GST)

GST activity was assessed in the liver cytosolic fractions as described by Habig et al. [34] using 1-chloro-2,4-dinitrobenzene (CDNB) (1 mM final concentration) as substrate in the presence of excess GSH (5 mM). The rate of CDNB conjugation was estimated by direct spectrophotometry at 340 nm for 3 min. The result was expressed as *μ*M GS-CDNB formed/min/mg protein.

### 2.9. Assay of Thiobarbituric Acid Reactive Substances (TBARS) Level in Liver

Lipid peroxidation products, namely, malondialdehyde (MDA) was estimated in liver microsomes assuming high PUFA content of microsomal membranes. The level of lipid peroxidation as measured by TBARS was determined according to the method of Buege and Aust [35]. Briefly, 1 mL of microsomal sample was mixed with 2 mL of TBA-TCA-HCl mixture thoroughly and heated for 15 min in a boiling water bath. After cooling, the flocculent precipitate was removed by centrifugation at 1,000 g for 10 min. The absorbance of the supernatant was determined at 535 nm and expressed in terms of nM MDA/mg protein.

### 2.10. Catalase Assay

Catalase activity was assayed following the procedure of Aebi [36] as modified by Kawamura [37]. A 5% homogenate was prepared in 50 mM phosphate buffer (pH 7.0) and centrifuged at 12,500 ×g for 30 min at 4°C. The supernatant or the peroxisome-rich fraction was used as the sample. The sample (20 *μ*L) was added to 980 *μ*L of an assay buffer containing 50 mM Tris-HCl (pH 8.0), 9 mM H_2_O_2_, and 0.25 mM EDTA to constitute the assay volume of 1 mL. The decrease in ΔOD/min of that assay mixture was recorded at 240 nm for 1 min. The results were expressed as unit catalase activity/mg protein. 

### 2.11. Western Blot Analysis

#### 2.11.1. Sample Preparation

Whole cell protein extracts and Western blot analysis were performed as previously described [38]. Liver homogenates (10% w/v) were prepared in 50 mM phosphate buffer (pH 7.5) and centrifuged at 10,000 g for 20 min. The cytosolic supernatant was collected very carefully, and the protein content of the sample was measured following the method of Lowry et al. [39].

#### 2.11.2. Methods for Western Blotting

Protein (60 *μ*g) from the lysates of control and treated cells was resolved on 10% SDS-PAGE at a constant voltage (60 V) for 2.5 h and then blotted onto a polyvinylidene fluoride (PVDF) membrane using a semidry trans blot apparatus (Bio-Rad Trans Blot SD Cell, USA). The membranes were first incubated with primary antibodies at a dilution of 1 : 1000 overnight at 4°C, followed by 2 h incubation with corresponding ALP-conjugated secondary antibodies at 1 : 2000 (Sigma) dilutions with continuous rocking. The immunoreactive bands were detected by using 5-bromo-4chloro-3-indolylphosphate/nitroblue tetrazolium (BCIP/NBT). Densitometric quantification was done by ImageJ (NIH) software.

### 2.12. Total RNA Extraction and RT-PCR Analysis

Total RNA from liver tissues were extracted using TRI reagent. Equal amounts of RNA (5 *μ*g) were reverse transcribed into cDNA using the RevertAid reverse transcriptase (Fermentas) following manufacturers protocol for RT-PCR. The PCR was performed following the procedure as per the manufacturer's instruction for 35 cycles. All test samples were amplified simultaneously from equal volume of first strand cDNA with the particular primer pair using a master PCR mix. PCR reactions were run in a programmable thermal cycler (GeneAmp 9700, ABI) with simultaneous NTC (no template control). *β*-actin was amplified simultaneously as an internal control. Specific primers for Nrf2 and *β*-actin for PCR amplification are Nrf2 forward 5′-TCTCCTCGCTGGAAAAAGAA-3′ Nrf2 reverse 5′-AATGTGCTGGCTGTGCTTTA-3′; *β*-actin forward 5′-TGGAATCCTGTGGCATCCATGAAAC-3′  *β*-actin reverse 5′-TAAAACGCAGCTCAGTAACAGTCCG-3′ [40]. For amplification of Keap1, specific primer was designed using Primer 3 software, Keap1 forward 5′-GTACGCTGCGAGTCCGAGGT-3′ Keap1 reverse 5′-GCCATTGCTCGGGTTGTAGG-3′. The PCR products were run in 1% agarose gel and visualized in a gel documentation system (Gel Doc EZ Imager, Bio-Rad) after staining with ethidium bromide. The densitometric quantification was done using ImageJ (NIH) software. 

### 2.13. Hoechst 33342 Staining

Hepatocytes were isolated from blanched liver by a two-step collagenase (Sigma Aldrich, USA) digestion method [41]. The isolated hepatocytes from different treatment groups were washed with PBS, fixed with 3.7% para-formaldehyde solution at room temperature, stained with bisBenzimide H 33342 trihydrochloride (Hoechst 33342; 2 mg/mL), and visualized under fluorescence microscope (Dewinter, Italy) within 30 min of adding the stain.

### 2.14. EDXRF Measurement

Liver was dissected out carefully using stainless steel forceps and blotted free of blood. The samples were not washed to avoid leaching of soluble elements. Tissues were freeze dried in a lyophilizer after fixing in liquid nitrogen and made into fine powder using mortar and pestle. About 150 mg pooled powdered samples were used to make into pellets (1 mm thick and 10 mm diameter) using a tabletop pelletiser (pressure: 100 to 110 kg/cm^2^ for 5 minutes).

The tissue samples were analyzed by Jordan Valley EX-3600 ED-XRF system. All measurements were carried out in vacuum using different filters (between the source and sample) for optimum detection of elements for 600 s. The X-rays detection was done by using liquid nitrogen cooled 12.5 mm^2^ Si (Li) semiconductor detectors with the resolution 148 eV at 5.9 KeV. The X-ray fluorescence spectra were quantitatively analyzed using Ex-Win software integrated with the system. Standardization of the procedure was done using NIST bovine liver standard SRM 1577b. 

### 2.15. Statistical Analysis

All assays were repeated at least three times. Data were analyzed by Student's *t*-test using the Sigma plot 8.0 statistical package. Differences between control and experimental group(s) with a value of *P* < 0.05 was considered as significant.

## 3. Results 

### 3.1. Mortality and Clinical Observations

All mice were examined daily for any clinical signs of toxicity. There was no death in both control and treatment groups, and no clinical symptoms were found to appear in any of the treatment groups.

### 3.2. Change in Body Weight and Water Consumption Rate

There was no significant difference in water consumption rate as well as change in body weight (weight gain) recorded between the control and treatment groups (data not shown). 

### 3.3. Organ to Body Weight Ratio

**Figure 1:**
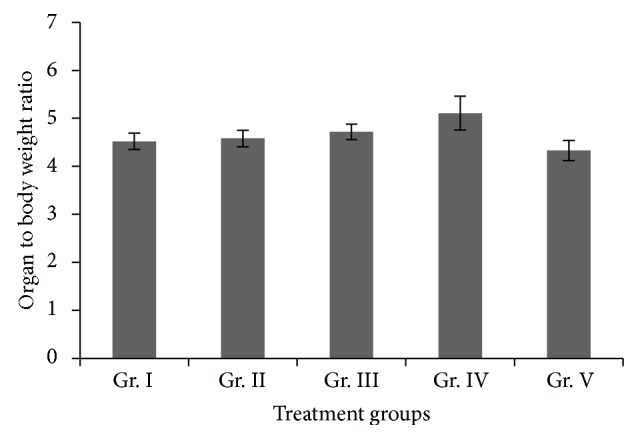
Organ to body weight ratio of liver in different groups of mice. Groups II, III, IV, and V were compared with group I. Values are expressed as mean ± SEM.

No significant difference in the organ to body weight ratio of liver of any treatment group with the control group was observed (Figure 1).

### 3.4. Histopathology

**Figure 2:**
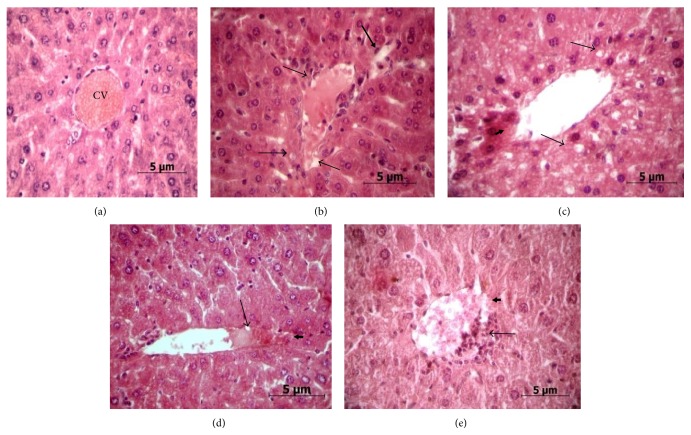
Changes in liver histology. Microphotographs of liver sections (6 *μ*m) stained with hematoxylin and eosin (HE). The original magnification: ×400. (a) Normal histological appearance of liver tissue of control mice, central vein (CV). (b) Group II: vacuolar degenerations (thin arrows), sinusoidal dilation (thick arrow), (c) Group III: disruption of epithelium lining (short thick arrows) of the central vein, vacuolar degenerations (thin arrows). (d) Group IV: vacuolar degeneration (thin arrow), loss of integrity in epithelium lining of the central vein (short thick arrow), and loss of typical hepatic cords organization. (e) Group V: extensive degeneration of epithelial lining of the central vein (thick arrow), loss of typical hepatic cord organization, hepatocellular degradation, and infiltration of the nucleus into the central vein (thin arrow).

Liver appeared normal and healthy (Figure 2(a)) in the control group of mice. Disorganization of hepatic parenchyma and disruption in the epithelial lining of the central vein and vacuolar degeneration were commonly observed in all treatment groups. Liver in Group II mice demonstrated sinusoidal dilation and vacuolar degeneration in the cytoplasm (Figure 2(b)). In group III mice (treated with 2 ppm of As_2_O_3_), extensive vacuolar degeneration and loss of integrity in the epithelial lining of the central vein were found (Figure 2(c)). Loss of typical organization of hepatic cord and vacuolar degeneration were seen in group IV mice (treated with 4 ppm of As_2_O_3_ for 30 days) (Figure 2(d)). In group V mice, treated with 4 ppm of As_2_O_3_ for 90 days, extensive degeneration of epithelial lining of the central vein (thick arrow), loss of typical hepatic cord organization, hepatocellular degradation, and infiltration of the nucleus (thin arrows) into the central vein were prominent (Figure 2(e)).

### 3.5. Liver Function Test

**Figure 3:**
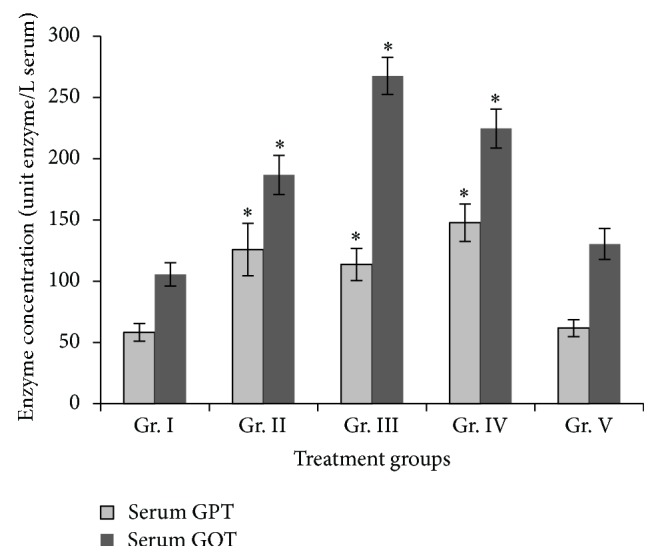
SGPT and SGOT activity (unit enzyme/L of serum) in the serum of different groups of mice exposed to different doses of As_2_O_3_. Groups II, III, IV, and V were compared with group I. Values are expressed as mean ± SEM. ∗Values are statistically significant at (*P* < 0.05).

An overall increase was noted in the serum GOT (AST) and GPT (ALT) levels in all treatment groups against control (Figure 3). SGOT level increased significantly in group II (77.94%), group III (153.79%), and group IV (113.04%) mice. SGPT level also increased significantly in group II (115.96%), group III (95.09%), and group IV (153.73).

### 3.6. GSH and GST Response and TBARS Production

**Figure 4:**
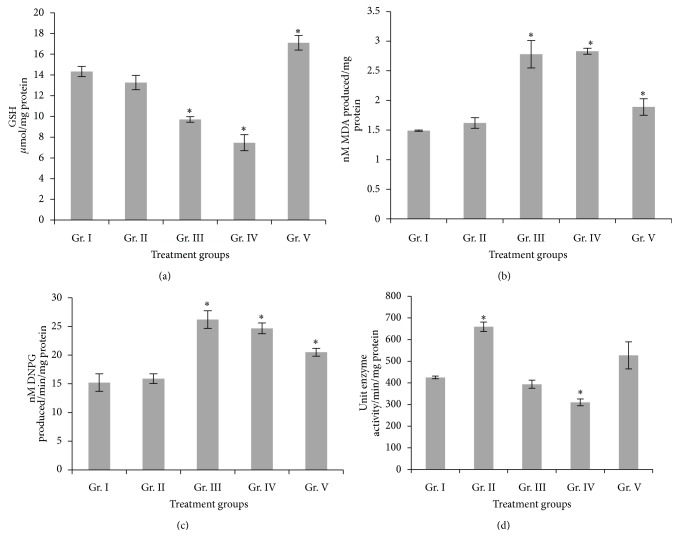
(a) GSH content, (b) MDA production, (c) GST activity, (d) catalase activity in liver of different groups of mice exposed to different doses of As_2_O_3_. Groups II, III, IV, and V were compared with Group I. Values are expressed as mean ± SEM. ∗Values are statistically significant at (*P* < 0.05).

A dose dependent decrease in GSH level was recorded in all the 30 days treatment groups of mice, and the decrease was significant in group III (32.29%) and group IV (47.9%) against control. In group V mice, after 4 ppm of As_2_O_3_ treatment for 90 days, GSH level recovered which increased significantly against control mice (19.31%) (Figure 4(a)). TBARS increased significantly in group III (86.56%), group IV (89.93%), and group V (26.84%) mice. GST activity also depicted a significant increase in group III (72.19%), group IV (62.13%), and group V (34.78%) mice (Figures 4(b) and 4(c)).

### 3.7. Catalase Activity

Catalase activity increased significantly in group II (55%) and thereafter gradually decreased with subsequent higher doses in group III (7.44%) and group IV (27.09%) mice against control. In group V, catalase activity showed an increase, though the increase was not significant compared to the control (Figure 4(d)).

### 3.8. Heat Shock Protein (Hsp) 70 Expression

**Figure 5:**
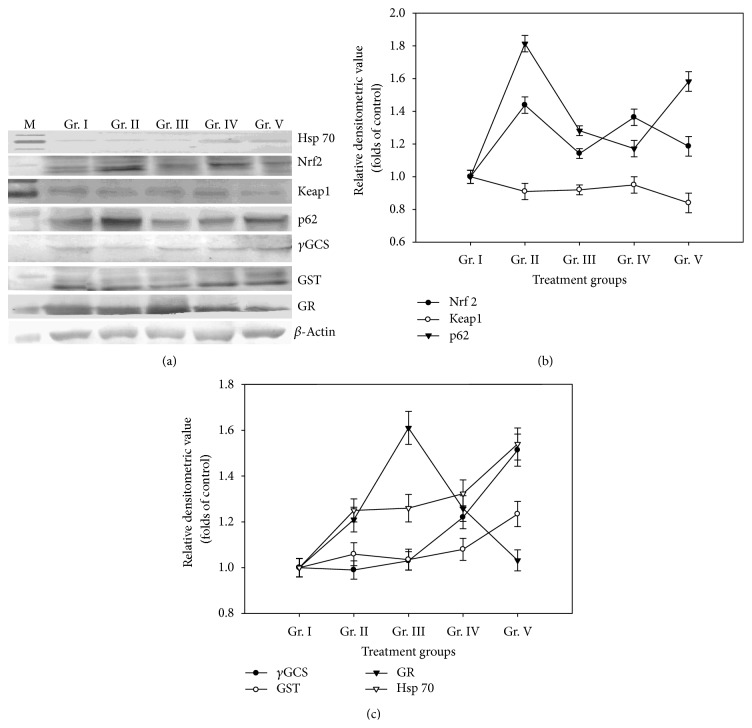
(a) Western blotting, (b) densitometric analysis of the Nrf2 protein, Keap1 protein, and p62 protein profile and (c) densitometric analysis of *γ*GCS protein, GST protein, GR protein, and Hsp 70 protein profile of liver of mice treated with different doses of As_2_O_3_.

Hsp 70 profile showed increasing pattern of expression against control in all the treatment groups. Expression increased 1.25-folds, 1.26-folds, 1.32-folds, and 1.54-folds in group II, III, IV, and V mice, respectively, as against the control group (Figures 5(a) and 5(c)).

### 3.9. Nrf2, Keap1, and p62 Protein Expression

Nrf2 protein levels were detected in whole cell lysates as this can give an idea of relative Nrf2 levels in the nuclear fractions of arsenic treated cells [20, 42, 43]. We observed the induction of Nrf2 protein for different doses of arsenic in all the treatment groups, but the increase in the Nrf2 protein level was not consistent with the increasing doses of arsenic treatment. The maximum level of Nrf2 protein was recorded in group II (1.44-fold of control) mice, which was found to decrease in group III (1.14-fold) mice. Further in group IV mice, the level of Nrf2 protein increased to 1.36-folds of the control again showing a decreasing pattern in group V (1.18-fold) mice (Figures 5(a) and 5(b)). Keap1 protein expression decreased in all the treatment groups compared to the control group. Lowest level of Keap1 protein was found in group V (Figures 5(a) and 5(b)) mice. p62 protein expression was also low in the control group, while elevated levels of protein expression were observed in all the treatment groups though the increase in protein level was not dose dependent. The highest level of p62 protein was observed in group II (1.8-fold of control) followed by group III and group IV mice where it decreased gradually by 1.3-fold and 1.2-fold respectively. In group V mice, however, an elevation of 1.6 folds was recorded (Figures 5(a) and 5(b)).

### 3.10. *γ*GCS Protein, GST and GR Protein Expression

*γ*GCS protein level in group II mice showed a slight decrease as compared to the control group, while the protein level increased continuously in the subsequent groups by 1.03-fold, 1.22-fold and 1.51-fold in group III, IV, and V mice, respectively, against control (Figures 5(a) and 5(c)). GST protein expression increased in all treatment groups with the highest level recorded in group V (1.23-fold) (Figures 5(a) and 5(c)). Expression of GR protein also showed an increasing trend in group II, and III whereas in group IV and V mice the level gradually decreased and in group V mice, it reached almost the control level (Figures 5(a) and 5(c)).

### 3.11. Nrf2 and Keap1 mRNA Expression

**Figure 6:**
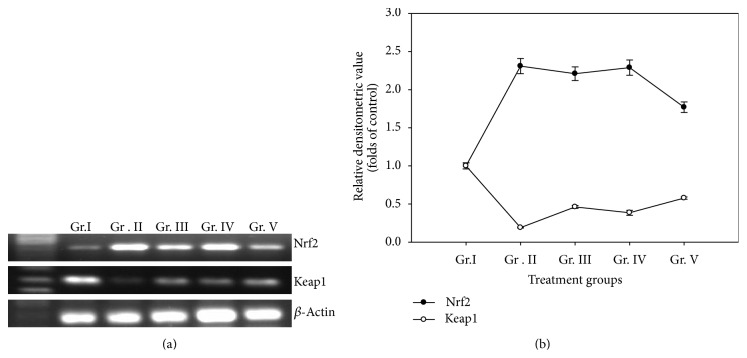
(a) Nrf2 and Keap1 mRNA expressions in liver. (b) Densitometric analysis of Nrf2 mRNA, Keap1 mRNA in liver of mice treated with different doses of As_2_O_3_.

Expression of Nrf2 mRNA increased in all the treatment groups compared to the control group. Expression increased by 2.31-fold, 2.21-fold and 2.29-fold in group II, group III, and group IV mice, respectively, against control group of mice. In group V (4 ppm of As_2_O_3_ treated for 90 days), decrement of the expression was noteworthy against the 30 days treatment groups (Figures 6(a) and 6(b)), though the level was still higher than control (1.77-fold). Keap1 mRNA level decreased in all treatment groups. Keap1 mRNA level detected was 0.81-fold that of control in group II, 0.54 folds of the control in group III, 0.61 folds of the control in group IV and 0.42-fold of control in group V mice (Figures 6(a) and 6(b)). 

### 3.12. Detection of Apoptosis by Hoechst 33342

**Figure 7:**
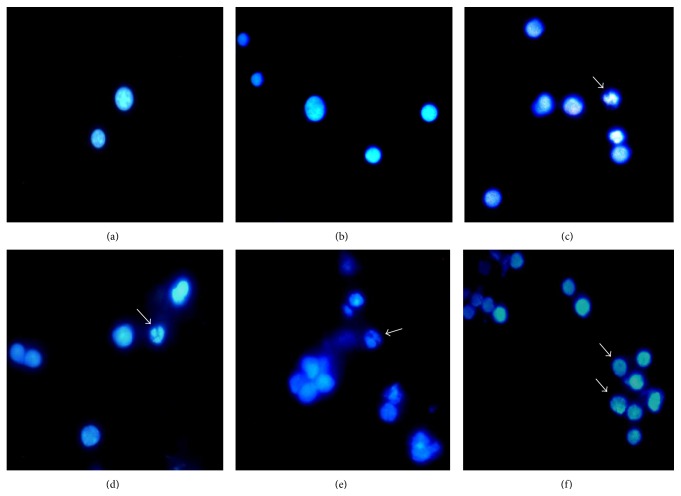
Mouse hepatocytes showing apoptosis (arrow) by Hoechst 33342 staining; (a), (b) control; (c) 0.4 ppm, (d) 2 ppm, and (e) 4 ppm As_2_O_3_ treatment for 30 days; (f) 4 ppm arsenic trioxide treatment for 90 days.

The hepatocytes of mice in all the treatment groups exhibited condensed and fragmented nuclei upon staining with Hoechst 33342, which is an indicator of possible apoptotic cell death (Figure 7).

### 3.13. Modulation of Elements

Concentration of Mg showed an increasing trend in all treatment groups and the increase was significant in group III, and group IV mice. Group II mice showed significant increase and decrease, respectively, for Cu and Zn, whereas significant depletion in Se level was observed in group IV mice. Iron concentration increased in group II, group III and group IV with the highest level were found in group IV mice. In group V mice, a reduction in the iron concentration was observed reaching almost the control value (Table 1).

## 4. Discussion

According to WHO guideline, the permissible limit of arsenic in drinking water is 10 ppb. In some states in USA and China, people are exposed to more than 1 ppm of As through their drinking water. Higher arsenic contamination is mainly found in ground water which is the most common source of drinking water. The chronic exposure is the main cause of arsenic induced cancer development in human population, and the mechanism of chronic arsenic toxicity is not fully known. Therefore, in our present experiment, mice were treated with As through drinking water. According to Singh et al. [44], arsenic administration decreased body weight gain in a dose dependant manner. Santra et al. [45] have reported significant increase in the liver weight of BALB/c mice after 12 months of arsenic treatment, whereas in the present study there was no significant change in body weight gain, rate of water consumption, or the organ to body weight ratio of liver in any of the treatment groups. 

Liver is an important organ for various metabolic pathways and effect of any chemical or xenobiotic appears primarily in the liver. In case of arsenic metabolism, liver is the main site of arsenic methylation [46]. The most common way of assertion of liver damage is to determine two pathophysiological enzymes, serum glutamate pyruvate transaminase (SGPT) and serum glutamate oxaloacetate transaminase (SGOT). In our findings, there was a distinct pattern of increased SGOT and SGPT levels in all the treatment groups treated for 30 days. In case of As_2_O_3_ (4 ppm) treatment for 90 days, however, reversal in the activities of both the enzymes occurred, almost reaching basal level. The reason for such reversal is difficult to explain since histopathological alterations as well as induction of apoptosis was observed in the same treatment group. The increased SGOT and SGPT levels in rat after 45 days treatment of 10 ppm sodium arsenite was also described by Tandan et al. [47], whereas Santra et al. [45] reported increased SGOT and SGPT levels after 12 months of arsenic treatment at 3.2 ppm in mice. Liver alteration in histopathological architecture was evident mainly in the form of loss of integrity of central vein, loss of hepatic cord organization, and dose and time dependent vacuolation which corroborates with earlier studies [45, 48, 49]. These studies indicate that chronic exposure to different inorganic arsenic compounds (arsenite, arsenic trioxide, or arsenate) produces characteristic pathology in the liver, including fatty infiltration, liver degeneration, inflammatory cell infiltration, and focal necrosis. Das Neves et al. [50] have shown that even acute exposure of sodium arsenite at a dose of 10 mg/kg body weight (intraperitoneally) for only 90 min could produce various histopathological alterations in liver. 

In the present study, Hoechst 33342 stained hepatocytes clearly indicated fragmented nuclei in all the treatment groups as a mark of apoptosis as reported earlier in fish liver [17].

Oxidative stress is a relatively new theory in assessment of arsenic toxicity [51, 52]. Arsenic causes oxidative stress by producing reactive oxygen species [20, 53, 54] which damage proteins. Due to lipophilic nature, arsenic also attaches to lipid thereby increasing the rate of lipid peroxidation [55]. Cellular GSH plays an important role in mitigating arsenic induced oxidative stress. Arsenic, being a strong electrophile, predominantly binds with nucleophilic SH group of GSH. Thus consequent depletion of GSH may alter the redox status of the cell and present a stressful and toxic situation. According to Bashir et al. [56], acute treatment of sodium arsenite at doses of 6.3 mg/kg, 10.5 mg/kg, and 12.6 mg/kg of body weight for 24 h significantly decreased GSH content in liver for all doses tested. On the other hand, a significant increase in lipid peroxidation and glutathione peroxidase activity along with significant decrease in the activity of GST, catalase, and superoxide dismutase was observed at 10.5 mg/kg and 12.6 mg/kg of As treatment. Santra et al. [45] reported that chronic exposure to 3.2 ppm of arsenic significantly depleted GSH after 6 months of exposure, catalase activity significantly decreased after 9 months, and GST activity significantly reduced at 12 and 15 months of arsenic exposure. In our study, GSH level showed decreasing trend in all the 30 days treatment groups with highest depletion recorded at 4 ppm of As_2_O_3_ treatment for 30 days, while after 90 days treatment with the same dose, the GSH level increased significantly with respect to both control and also the 30 days treatment groups. This shows the upregulation of GSH against long term arsenic treatment which is due to its adaptive response to oxidative stress [20]. However, a reverse trend was observed in TBARS production and GST activity in liver; in group II (0.4 ppm As_2_O_3_ treatment for 30 days), there was almost no change in TBARS and GST level against the control group, while in group III and group IV, levels of both TBARS and GST activity increased significantly. Interestingly, in group V (90 days treatment with 4 ppm As_2_O_3_) both TBARS and GST level decreased significantly with respect to group IV, that is, 4 ppm As_2_O_3_ for 30 days, though the levels were still significantly higher than the control group. The increased GST activity might be responsible for the reduction in GSH while protecting against the arsenic-induced oxidative stress. Catalase activity increased significantly in group II mice with respect to control, while at subsequent higher doses that is, 2 ppm and 4 ppm As_2_O_3_ for 30 days, the activity decreased and highest rate of depletion was observed after 4 ppm As_2_O_3_ treatment for 30 days. Further, in group V (4 ppm As_2_O_3_ for 90 days), the recovery in the catalase activity was observed and it increased significantly when compared to group IV (4 ppm As_2_O_3_ treatment for 30 days). According to Mittal and Flora [57], catalase activity significantly decreased in liver and kidney with respect to control after treatment with 100 ppm sodium meta-arsenite in drinking water for 8 weeks.

Trace elements play an important role in maintaining normal homeostasis of the body. Oxidative stress results from changes in the levels of trace elements. It has been reported that increased Mg level is associated with the increased production of MDA in dementia patients [58]. Our result also showed an increasing pattern of Mg concentration along with increase in liver TBARS level. 

Zn is an essential element, required for growth and normal development, and is a constituent of more than 200 enzymes, one of which is a Cu/Zn superoxide dismutase (Cu/Zn SOD). Cu/Zn SOD is a powerful antioxidant which transforms free radical O_2_^•^ to H_2_O_2_, therefore reducing the risk of formation of highly reactive hydroxyl radical HO^•^. Cu is also an integral part of Cu/Zn SOD enzyme. Decreased Zn and Cu levels in tissues may result in reduction of Cu/Zn SOD activity and subsequently accelerate the process of cell aging and death via oxidative damage [59]. However, free or incorrectly bound Cu^+2^ can catalyze the generation of the most damaging radicals, such as HO^•^, resulting in a chemical modification of the protein, alterations in protein structure and solubility, and oxidative damage to surrounding tissue [60]. Our findings also indicate significant modulation of Cu and Zn level in liver of group II mice. 

Selenium (Se) is a well known antagonist of arsenic toxicity [61]. The most probable cause of such a protective effect of selenium is due to its ability to upregulate antioxidant enzymes like GSH peroxidase and thioredoxin reductase which protects against arsenic induced oxidative damage [62]. Decrement in selenium level after As_2_O_3_ treatment was reported by Molin et al. [63, 64]. In the present study, Se level in liver decreased dose dependently recording maximum depletion at 4 ppm of As_2_O_3_ for 30 days but in the group, treated for 90 days with 4 ppm As_2_O_3_, Se level increased and almost reached the normal level.

Iron (Fe) catalyses the formation of reactive oxygen species through the Fenton and Haber-Weiss reactions [65], which generate highly toxic hydroxyl radical and cause lipid peroxidation [66]. According to Ahmad et al. [67], body iron stores (serum ferritin and transferrin saturation) in the body can be used as an early investigative tool for assessing the oxidative stress in coronary heart disease. In our study, iron level increased in all the treatment groups except in group V, where the iron concentration is almost same to that of the control group.

Heat shock proteins (Hsps) are expressed in tissues in response to a harmful stress situation or adverse life conditions. Roy and Bhattacharya [17] first reported about increased Hsp 70 in the liver of *Channa punctatus*, a fresh water teleost. Similar induction of Hsp 70 protein was also found in the present study in all the treatment groups which corroborates earlier finding.

Mammalian cells cope with xenobiotics by adaptive defense mechanisms to maintain cellular homeostasis and physiological functions [20]. Nrf2/Keap1/ARE driven target gene system is one such mechanism [28]. In a recent study, Li et al. [20] reported induction of Nrf2 protein in Chang human hepatocytes. They observed that 5 and 10 *μ*mol/L of sodium arsenite increased the Nrf2 protein levels significantly at 6 and 12 h and a decrease thereafter. They also observed that 5 to 25 *μ*mol/L of arsenic could increase the Nrf2 protein levels significantly, but 50 *μ*mol/L of arsenic did not have similar effect. They opined that this resulted due to the cytotoxicity caused at higher dose of arsenic.

**Figure 8:**
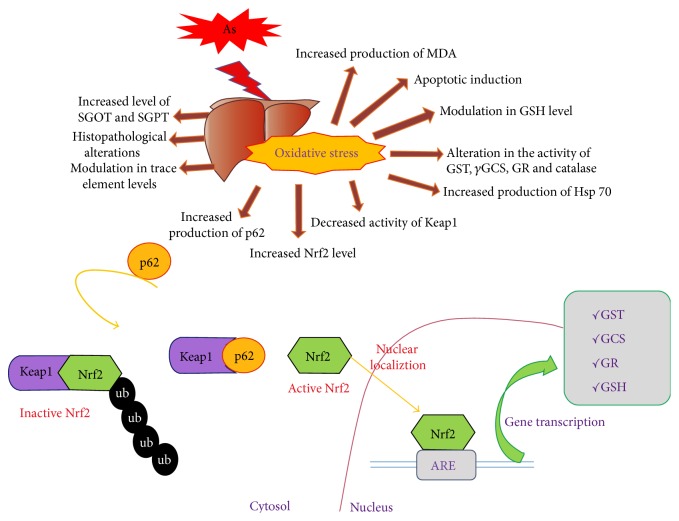
Schematic representation of the arsenic induced liver damages and induction of gene expression through the Keap1-Nrf2-ARE signaling pathway.

Our observation on the Nrf2 protein induction also did not show a pattern of consistent increase within 30 days treatment groups. The maximum induction was observed at 0.4 ppm arsenic treatment, which decreased thereafter. Nrf2 protein level after 30 days treatment with 4 ppm of arsenic was higher than that of 90 days treatment group supporting the theory of adaptive response mechanism [20]. The pattern of Nrf2 mRNA level also matched with the corresponding protein level. We also monitored the expression patterns of two important proteins, Keap1 and p62, which are closely related to the expression and transfer of Nrf2 protein from cytoplasm to nucleus and the activation of ARE driven genes like *γ*GCS, GR, and GST involved in glutathione metabolism. Keap1 (Kelch-like ECH-associated protein 1) plays a vital role in the localization of Nrf2 protein within cytoplasm by binding and thereby inhibiting the Nrf2 activity and p62 plays its role by docking the Keap1 protein through a motif called Keap1 interacting region (KIR) thereby blocking binding between Keap1 and Nrf2. As a result, Nrf2 protein is separated from Keap1 following its migration to the nucleus. Interestingly, induction of p62 results from oxidative stress and is mediated by Nrf2 which binds to the ARE containing cis-element of p62. Therefore p62/SQSTM1 (sequestosome 1) is a target gene for Nrf2 which creates a positive feedback loop by inducing ARE driven gene transcription [68]. In the present study we report synchronization of Keap1 and p62 levels with the Nrf2 protein level accompanied by induction of the downstream genes, GCS, GR, and GST, involved in GSH metabolic pathway. The role of Nrf2 signaling pathway in cellular protection after arsenic exposure is schematically represented in the Figure 8. To the best of our knowledge, this is the first report on the effect of arsenic on Keap1-P62-Nrf2 signaling pathway with respect to expression of the ARE driven genes related to GSH metabolic pathway in mouse liver *in vivo*. The expression pattern of the GCS, GR, and GST are in agreement with the Nrf2 mediated antioxidative and adaptive response mechanisms against arsenic induced damages in liver.

## 5. Conclusion

The present study clearly indicates that treatment of arsenic trioxide through drinking water in mice *in vivo* induces hepatotoxicity as evidenced by oxidative stress and histopathological changes with concomitant effect on normal liver function, and activates the Keap1/p62/Nrf2 signaling pathway leading to activation of downstream ARE driven genes related to GSH metabolism, involved in protecting cells against arsenic induced oxidative stress, and activates the adaptive response mechanism. The alterations in the expressions of the ARE-driven genes might help in understanding the mechanism of chronic arsenic induced hepatotoxicity in mammals including human beings facing serious threats in severe arsenic endemic regions all over the world.

## Figures and Tables

**Table 1:** Concentration of elements (mg/kg) in whole-liver tissue following *in vivo* administration of different concentrations of As_2_O_3_ (ppm) in the drinking water of the mice for 30 and 90 days (values are mean ± SEM).

Elements	Group I	Group II	Group III	Group IV	Group V
Mg	354.45 ± 54.17	547.29 ± 58.88	678.79 ± 21.13^*^	578.21 ± 42.46^*^	461.43 ± 29.90
Cu	15.41 ± 0.23	17.49 ± 0.73^*^	14.54 ± 0.72	14.44 ± 0.47	14.68 ± 0.59
Zn	107.55 ± 1.57	97.63 ± 3.97^*^	103.77 ± 1.64	107.62 ± 3.35	102.26 ± 4.71
Se	14.19 ± 1.34	13.25 ± 0.55	12.52 ± 1.89	8.43 ± 0.74^*^	14.09 ± 1.13
Fe	503.65 ± 38.66	610.29 ± 45.99	567.82 ± 22.97	734.21 ± 44.94^*^	506.89 ± 28.22

^*^Values are statistically significant compared to control at (*P* < 0.05).
